# Spontaneous Iliac Arteriovenous Fistula Leading to High-Output Heart Failure and Cardiac Arrest

**DOI:** 10.7759/cureus.51876

**Published:** 2024-01-08

**Authors:** J. Maxwell Piraneo, Russell Mordecai, James Espinosa, Alan Lucerna

**Affiliations:** 1 Emergency Medicine, Jefferson Health, Stratford, USA

**Keywords:** cardiac arrest, emergency department management, high-output heart failure, iliac aneurysm with rupture, iliac arteriovenous fistula

## Abstract

We report a case of a 70-year-old male who complained to family members of the sudden onset of groin pain. He then collapsed, and emergency medical services were called. The patient arrived at the ED with a return of spontaneous circulation after cardiac arrest. The patient was diagnosed with a spontaneous iliac arteriovenous (AV) fistula secondary to aneurysmal rupture. This is a rare but potentially life-threatening condition that can result in high-output heart failure and, as described here, cardiac arrest. The differential diagnosis of groin pain is vast, but in the setting of cardiac arrest, vascular causes must be considered. Treatment is most often operative intervention, as was the case with the patient presented. It is predictable that as the population ages and invasive vascular surgeries become more common, the incidence of iliac AV fistulas will increase, resulting in more presentations of high-output heart failure or cardiac arrest in the emergency department.

## Introduction

High-output heart failure is a potentially life-threatening condition that can lead to cardiac arrest. The most common causes of this condition are obesity, liver disease, arteriovenous (AV) shunts, lung disease, and myeloproliferative disorders [1]. Here, we describe an unusual case of cardiac arrest as a consequence of high-output heart failure, secondary to rupture of an iliac artery aneurysm into the common iliac vein, with AV fistula formation. This article was previously presented in poster format at the Rowan-Virtua SOM Research Day on May 4, 2023.

## Case presentation

A 70-year-old male presented to the ED by emergency medical services (EMS) after a cardiac arrest. EMS found the patient at home in pulseless electrical activity (PEA). Cardiopulmonary resuscitation was performed with one dose of epinephrine administered. The patient was intubated in the field. The patient regained spontaneous circulation and arrived at the ED, unresponsive but with a pulse. The patient’s wife reported that the patient had been complaining of groin pain for a four-hour duration. He reportedly walked outside when his wife heard him collapse on the ground. The patient’s wife stated that the patient had no history of recent illnesses and that the patient did not regularly see any physicians or take any medications.

On presentation, his vital signs were as follows: blood pressure 97/63 mmHG, heart rate 160 beats per minute, temperature 94.3 Fahrenheit rectally, respiratory rate of 22 breaths per minute, and pulse oximetry on 100% oxygen on a ventilator. A physical examination revealed an ill-appearing, unresponsive male. Pupils were 3 mm in size and were equal, round, and reactive to light. His heart examination noted an irregularly irregular rhythm. Breath sounds were coarse bilaterally with symmetric chest expansion. His abdomen was soft with normal bowel sounds, and no distention was noted. The neurologic exam was limited due to unresponsiveness and a GCS of 3. The physical exam was otherwise unremarkable.

**Figure 1:**
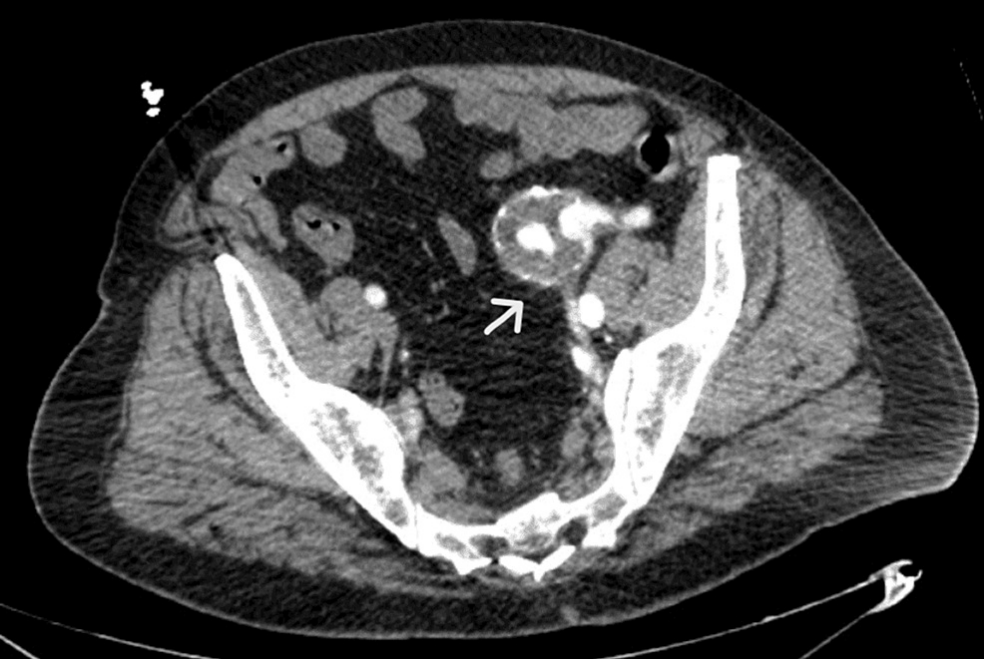
CT with IV contrast demonstrating an iliac artery aneurysm with an AV fistula (arrow)

An ECG revealed atrial fibrillation at 138 beats per minute with nonspecific ST-T abnormalities. A high-sensitivity troponin was elevated at a level of 469 ng/L. The lactate result was elevated at 5.6 mmol/L. Arterial blood gas results were as follows: pH 7.04, PCO2 of 42 mmHg, pO2 mmHg of 400, and bicarbonate of 11 mEq/L. The basic metabolic panel was significant for potassium at 2.5 mmol/L and was otherwise within normal limits. The white blood cell count was 16.3 cells/mm3, with an elevated hemoglobin of 16.4 g/dL. The remaining lab work, including hepatic enzymes, lipase, ethanol, salicylate, and creatine kinase, was all unremarkable. A CT scan with IV contrast was significant for a 7 cm left common iliac artery aneurysm with rupture into the common iliac vein with AV fistula communication between the two vessels (Figure 1).

**Figure 2:**
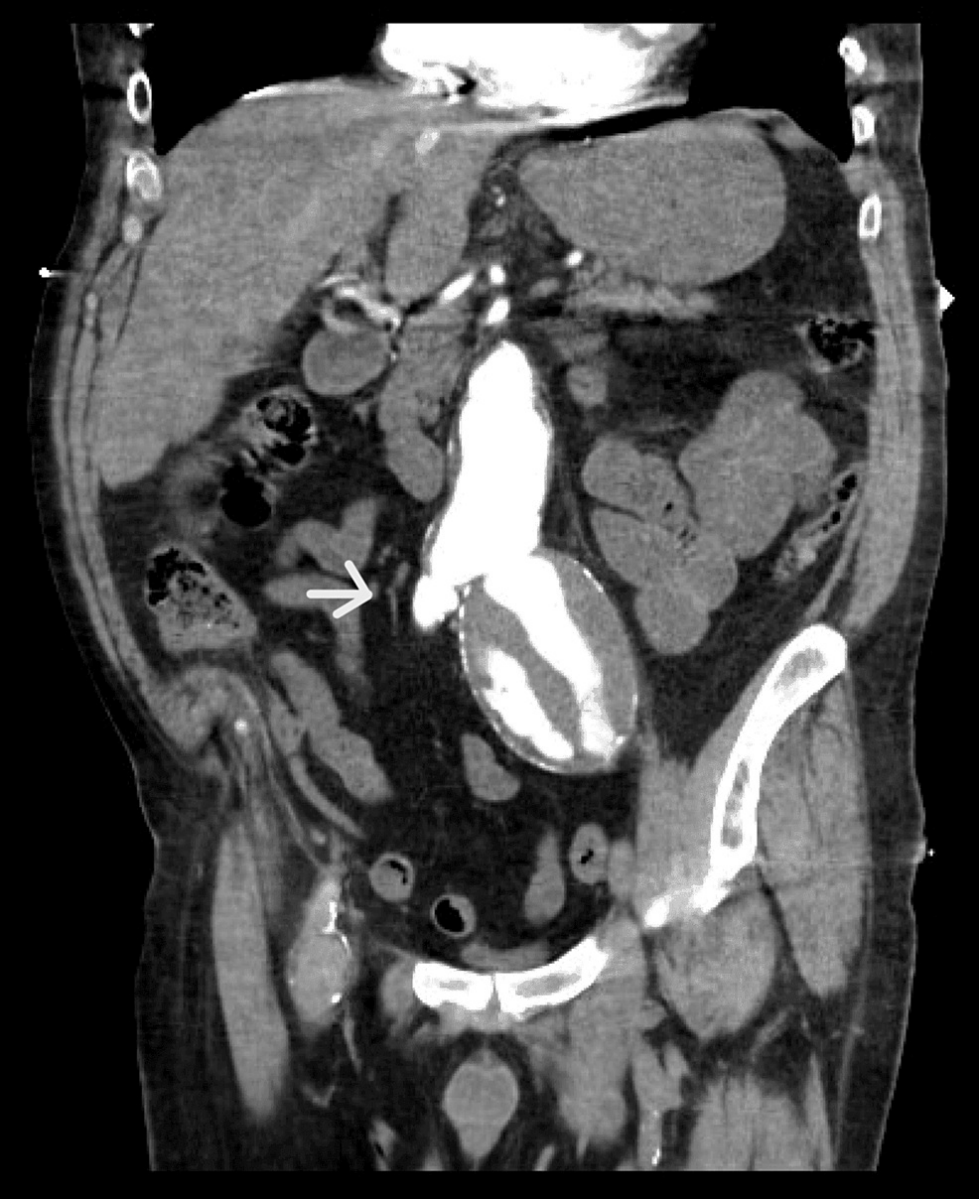
CT with IV contrast demonstrating contrast filling the iliac artery and vein (arrow)

The CT scan showed a contrast filling the iliac artery and vein (Figure 2).

**Figure 3:**
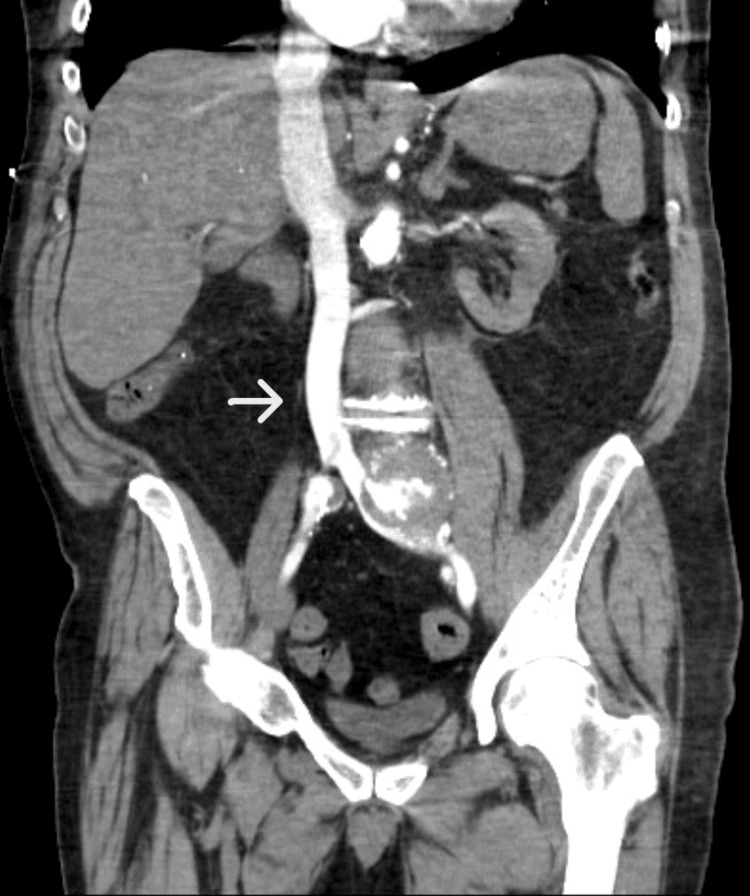
CT with IV contrast demonstrating reflux of contrast in the inferior vena cava secondary to fistula (arrow)

Reflux of contrast into the inferior vena cava secondary to the fistula was seen on the CT scan (Figure 3).

There was no evidence of acute pulmonary emboli or pleural effusions. A CT scan of the head was unremarkable. A repeat ECG showed atrial fibrillation at 171 beats per minute with a rightward axis with ST depressions in leads V4 through V6, new ST depressions in leads I and aVL, and new ST elevations in leads V1 and V2. Medications administered included a normal saline fluid bolus (one liter), potassium 20 mEq IV and 40 mEq via an orogastric tube, magnesium two grams IV, ceftriaxone one gram IV, and fentanyl 50 mcg IV. A Cardizem infusion was initiated at 5 mg/hr in order to manage the atrial fibrillation with rapid ventricular response.

A call was immediately placed to the vascular surgery team. The patient was transported by helicopter for emergent endovascular aneurysmal repair. At the receiving facility, the patient was immediately brought to the operating suite, where he again sustained a PEA cardiac arrest. Despite the insertion of a resuscitative endovascular balloon for aortic occlusion proximal to the AV fistula, the patient did not regain spontaneous circulation with minimal cardiac electrical activity and was pronounced deceased.

## Discussion

Pathophysiology of high-output heart failure

High-output heart failure is a less common form of heart failure and is the result of the heart’s inability to keep up with metabolic demand. This is distinct from the more common forms of heart failure, which are a result of systolic or diastolic dysfunction. Patients with high-output heart failure have normal cardiac function with decreased vascular resistance, often a consequence of another underlying disease process. This condition has two main physiologic causes: an increase in oxygen consumption from increased metabolism, leading to an increase in the body’s demand for blood, and a decrease in systemic vascular resistance, often due to bypass of the arteriolar and capillary bed, leading to increased flow into the venous circulation and increased venous return [1]. The increase in cardiac output creates substantial myocardial stress, leading to heart failure.

Incidence and clinical presentation

AV shunt formation is among the most common reasons for high-output heart failure. AV fistula creation is most often encountered in patients with end-stage renal disease who require access to dialysis. Even among these patients, high-output heart failure is a rare complication [2]. Iliac AV fistula refers to the formation of an abnormal communication between the iliac artery and vein, often a result of operative or penetrating trauma or, less often, spontaneous aneurysmal rupture. Aneurysm of the iliac artery is the most common form of aneurysm after abdominal aortic aneurysms, which is itself often a consequence of prolonged and uncontrolled blood pressure elevation [3]. Complaints of lower abdominal or flank pain are common, with clinical features including an abdominal bruit, pulsatile mass, and edema. Iliac AV fistula is a rare diagnosis that can carry a mortality rate of up to 60% [4].

Diagnosis

Diagnosis of an iliac AV fistula can be made using angiography, duplex and color Doppler sonography, MRI, and CT. CT angiography is often considered a superior diagnostic tool for ED use, given that it is rapid, minimally invasive, accurate, and less operator-dependent and has sensitivities from 90 to 100% and specificities from 90 to 100% [5].

Management

Treatment options vary from temporary conservative management through compression therapy or more definitively through open or endovascular repair with vascular surgery [6]. The principal objectives of treatment are arterial repair and removal of venous communication to prevent further arterial dilation, rupture, and progression of sequelae such as high-output heart failure [7].

## Conclusions

Here, we report a case of cardiac arrest as a result of high-output heart failure due to an iliac AV fistula. High-output heart failure is a potentially life-threatening condition that can lead to cardiac arrest. The most common causes of this condition are obesity, liver disease, AV shunts, lung disease, and myeloproliferative disorders. The patient in this case developed an iliac AV fistula as a result of an iliac aneurysmal rupture, leading to high-output heart failure and subsequent cardiac arrest.
